# The Role of Energy Visualization in Addressing Energy Use: Insights from the eViz Project

**DOI:** 10.3389/fpsyg.2016.00092

**Published:** 2016-02-09

**Authors:** Sabine Pahl, Julie Goodhew, Christine Boomsma, Stephen R. J. Sheppard

**Affiliations:** ^1^School of Psychology, Plymouth UniversityPlymouth, UK; ^2^Collaborative for Advanced Landscape Planning, University of British ColumbiaVancouver, BC, Canada

**Keywords:** thermal imaging, visualization, behavior change, energy efficiency, retrofit

Energy has become an important topic for policy makers, industry, and householders globally (e.g., IEA-International Energy Agency, 2015). Changing the way we generate and use energy could make a huge contribution to reducing carbon emissions and help address climate change. There is also concern over energy security where energy is imported from other countries. Fluctuations in energy prices affect industry and householders and are linked to fuel poverty, especially in vulnerable households (Liddell and Morris, 2010).

Increasing energy efficiency has been hailed as a key solution to dealing with the energy issue (see www.eceee.org) but has perhaps not received as much attention as is warranted by its huge potential. This may be because energy efficiency is not one specific solution; rather many small changes and interrelated steps are required. This is contrary to big-ticket visions such as finding a novel non-carbon source of energy or other solely technological solutions, ranging from renewed investment in nuclear power to automating energy processes in buildings.

Thus, there seems to be a degree of conflict between focusing on technology, automation etc. and a more systems-focused approach that includes social factors such as people's attitudes, values, and behaviors. We think the way forward is to integrate technical and social approaches, especially where they intervene in people's daily lives. It is necessary and important to communicate and discuss energy issues with the wider public in order to find acceptable, effective and sustainable solutions, whether these are grid-related or about specific buildings. For example, building science colleagues have often complained (jokingly or not) how occupants are “messing up” after key energy-saving technology has been implemented in home or work contexts (e.g., see literature around the energy performance gap). Moreover, recent work has highlighted the phenomenon of techno-optimism whereby people tend to overestimate the success of new technologies (Clark et al., 2015). These wider considerations serve to situate our eViz project (eViz.org.uk) from the point of view of social scientists working on an issue traditionally dominated by “technical” experts and views.

Having said this, the energy field is burgeoning with new initiatives (e.g., new journal Energy Research and Social Science) and explicitly social science-led research projects (e.g., www.projectcharm.info/; www.eviz.org.uk; http://c-tech.cloudapp.net/). At the global level, the IEA is funding work on behavior change (www.ieadsm.org/ViewTask.aspx?ID=16&Task=24&Sort=0) and occupant behavior (annex66.org/) although the latter group is still dominated by technical experts. The debate around technical, social and integrated solutions leads to the question of how best to communicate the energy use embedded in people's everyday actions and familiar surroundings, with the ultimate aim of engaging consumers and change behavior. The traditionally hidden nature of energy has been observed as one barrier to energy awareness, and making energy information accessible is suggested to empower people (Darby, 2001; Parnell and Popovic Larsen, 2005). In line with this are vigorous calls for making energy visible, including several nations' commitments to add energy displays to every home.

The present paper reports on work from our ongoing eViz project focusing on energy visualization. At the time of writing we are in the final year of this 3.5 year project that integrates researchers from psychology, building science, data visualization, and computing. We investigate how to reduce energy demand in buildings by transforming people's understanding and behavior through energy visualization. The Psychology literature suggests energy visualization is crucial for at least four reasons.

First, making the invisible visible attracts people's attention (Gardner and Stern, 2002). Attention is important when communicating any topic to the public, but especially in the current context as energy use tends not to be people's primary goal in day-to-day life. Visual imagery has the ability to communicate messages quickly and powerfully and enables the conceptualisation of complex issues (Sheppard, 2001, 2005). Second, there is a strong link between emotions and visual images. Basic emotions evolved relatively early, before the development of higher level conscious processes such as language. As a result, emotions are more dependent on and respond quicker to information in visual, rather than textual, form (Holmes and Mathews, 2010). Third, taking together the ability of images to grab attention and evoke emotions, images can be described as “vivid” representations (Nisbett and Ross, 1980). Vivid information is linked to a greater “imageability” (Taylor and Thompson, 1982) and the generation of mental images. These mental representations that take the form of sensory images (Slovic et al., 1998; Andrade et al., 2012) are easily retrieved and facilitate the processing of the message (Smith and Shaffer, 2000). So, visual information can be internalized in the form of mental images which aid memory. Fourth, research in cognitive psychology has indicated that mental images have an important motivational role through their link with goals (cf. Kavanagh et al., 2005). Mental images connect to emotions and trigger related goals after being activated through internal or external cues. Further, new goals might be formed by generating new mental images (Conway et al., 2004). In sum, according to psychology research, images attract attention, evoke emotions, facilitate memory, and trigger (the development of) goals (see illustration in Figure 1). Furthermore, through these properties, images may support social processes and aid collective action by serving as a shared catalyst and reinforcement between energy users.

**Figure 1:**
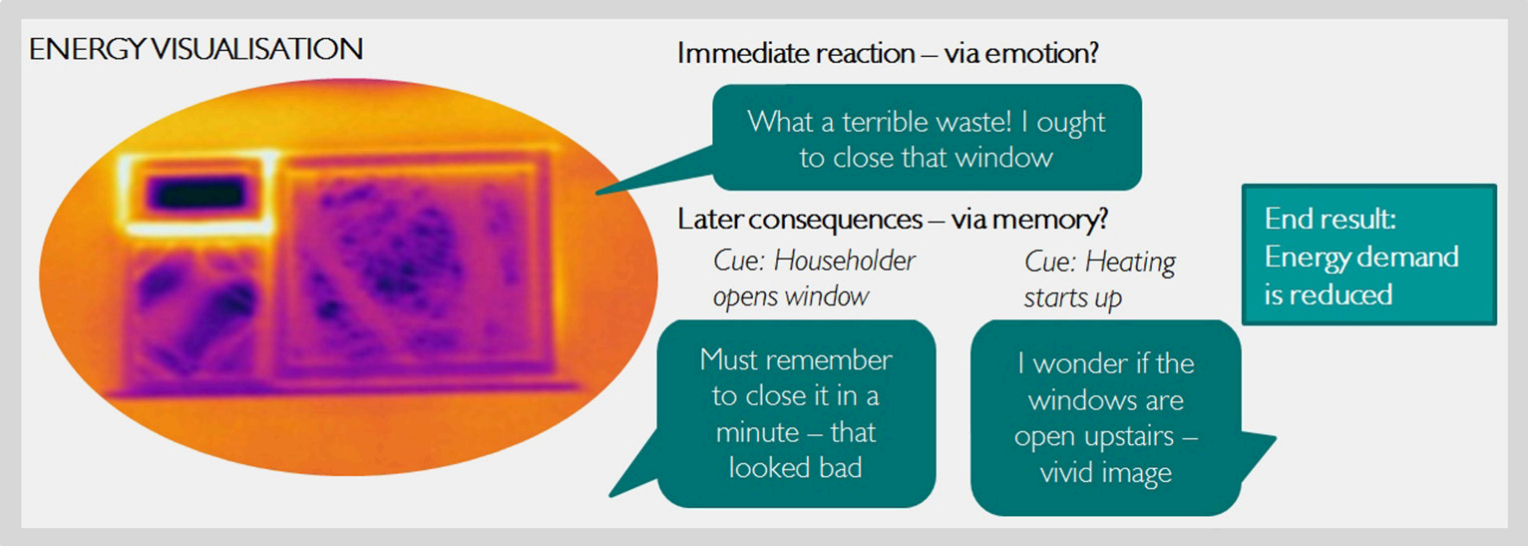
**Simplified illustration of suggested psychological processes around energy visualization using the example of a thermal image visualizing heat escaping through an open window in winter**. Bright yellow color indicates higher temperatures.

How can we apply these principles to the field of energy? There are different ways of visualizing energy. Energy displays in the home are one example but are they sufficiently attention-attracting, memorable, emotional, and meaningful? Buchanan et al. (2014) raise critical views on the ability of basic energy displays to engage consumers meaningfully and over long periods of time (see also Hargreaves et al., 2010). For the purpose of the present paper we will focus on research surrounding one specific tool: thermal imaging (see example in Figure 1).

Thermal images provide a visualization of heat through cameras detecting infrared radiation not normally visible to the human eye. Colors represent different temperatures and allow inferences where heat, and so energy, might be conserved in buildings. Unexpected areas of heat escaping from the building or cold air entering are made visible (Pearson, 2011; Fox et al., 2015). In sum, thermal imaging visualizations display information about energy use, which is normally invisible, in a colorful, vivid format.

The findings of four energy visualization studies provide insights into the role of thermal images in the field of domestic energy. The first small-scale study provided evidence that seeing thermal images increased energy efficiency actions. Householders were allocated to one of three conditions [thermal images of own home (*n* = 17), carbon footprint audit (*n* = 17), or control (*n* = 9)]. After one year, only the thermal image group reduced their carbon emissions from energy in the home, calculated from their household energy bills before and after the intervention. The behaviors they reported taking were directly related to the behaviors visible in the images (Goodhew et al., 2015a). In a second study, 87 householders received an energy audit. In addition, participants in the thermal image condition (*n* = 54) received thermal images of their own home but participants in the control condition (*n* = 33) did not receive any visualizations. Approximately 6 months later, householders who saw thermal images were almost five times more likely to report the installation of draught proofing measures compared to the control group (Goodhew et al., 2015a).

Having established evidence for a behavioral effect following exposure to thermal images, what do we know about the psychological process between energy visualizations and energy efficiency behavior? Householders see thermal images as highly desirable, and free voluntary thermal imaging offers trigger huge interest and immediate sign-ups (Goodhew et al., 2015b). An earlier exploratory qualitative study (Goodhew et al., 2009) examined the immediate reaction of ten householders at the moment they saw their tailored thermal images. Reactions were frequently (but not always) indicative of a fast attention capture. If householder attention was engaged successfully, this then led to an elaboration and reasoning process around homes, buildings, heat and energy, particularly in relation to householders' own behaviors. Participants' first response was often emotional and evaluative, e.g., “that looks dreadful!” This study provides first evidence that thermal images can attract attention to a complex set of issues that may be difficult to get across through alternative methods of communication (Sheppard, 2001, 2005; Midden et al., 2007). So, a key role of energy visualizations is in attracting attention, which is the first critical stage that can trigger an elaboration process taking the viewer from image to thoughts about energy and their own behavior (Goodhew et al., 2009).

In a fourth study, we investigated the importance of the tailoring element in the visualization. Householders (*N* = 233) were exposed either to thermal images of their own home (“tailored”), thermal images of other homes similar to theirs or a text only condition. Householders reported recalling thermal images more vividly than the text, irrespective of whether the images were of their own or another's home. However, own images were reported to be more intrusive: Householders said images of their own home “popped into their heads” more than images of others' homes. This suggests that thermal images led to more vivid mental images than text and that tailoring the presentation increased the intrusiveness of these mental images: they interfered with and interrupted normal thought processes. This is promising in terms of goal processes. If images are strong enough to keep appearing in memory, this dynamic could explain increased motivation and action. Finally, those who received the tailored thermal images looked at their information more often and reported sharing that information with more people (Boomsma et al., accepted).

Beyond these cognitive processes, householders who saw tailored images were most likely to change their plans for their homes and reported stronger energy related intentions along with a stronger belief that they would benefit from draught proofing measures. In sum, the role of thermal image visualizations may be in communicating an energy efficiency message with increased vividness and “imageability” which more readily connects to the viewer's goals. This can be achieved partly by providing thermal images of typical homes but tailoring the images to people's own homes enhances the effect.

To summarize, our research on thermal images as a way of visualizing energy suggests that they can promote energy efficiency behaviors. They can attract attention, invoke emotion and trigger vivid images in memory later on. The images also communicate the problem of energy wastage in an intuitive and specific manner, aiding comprehension. Through these processes, they can enhance motivation, establish, and promote energy efficiency goals and trigger action.

## Limitations and future work

Much remains to be investigated in the field of energy visualization. While we have good evidence that one type of energy visualization (thermal images) can change householder decisions and behavior, we know very little about the effects of presenting different aspects of thermal images (e.g., inside vs. outside perspectives). Also, this example has only been applied to heat escaping from homes in cold winter climates, always using English homes. Other contexts and uses are conceivable that vary in terms of energy prices (after earlier pilot work, a larger trial is now planned in Vancouver, Canada, for example). Thermal imaging could also be used to visualize energy use associated with air conditioning in hot climates and we are currently exploring this new application. Moreover, work is needed to investigate other energy visualizations, for example to optimize the displays associated with the smart meter rollout, in order to maximize benefit from these vast nation-wide investment programs. Finally, visualization could be combined with interactive engagement, e.g., through virtual reality where users can explore energy issues for themselves (Stone et al., 2014).

## Conclusions

Thermal images are one type of energy visualization that helps to engage householders in energy saving actions. We have summarized early evidence and psychological principles but further research is needed to test such integrative approaches that combine technical and social aspects intelligently. The area of energy visualization has huge potential that researchers are only beginning to exploit and can make an important contribution to the challenges surrounding climate change, energy security and fuel poverty.

## Author contributions

All authors listed, have made substantial, direct, and intellectual contribution to the work, and approved it for publication.

### Conflict of interest statement

The authors declare that the research was conducted in the absence of any commercial or financial relationships that could be construed as a potential conflict of interest.
